# Exercise training in patients with corrected tetralogy of Fallot: A protocol for systematic review and meta-analysis

**DOI:** 10.1097/MD.0000000000026108

**Published:** 2021-05-28

**Authors:** Ya-Qin Gong, Xiao-Yan Liu, Ping Zhi, Li-Na Wei, Fang-Fei Guo, Jin-Zhi Qian, Yun-Xia Wang, He-Li Dong

**Affiliations:** a Department of Cardiovascular Surgery, Gansu Provincial Hospital; b Congenital Heart Disease Diagnosis and Treatment Gansu Province International Science and Technology Cooperation Base, Lanzhou; c Department of Thoracic Surgery, The First Hospital of Longnan City, Longnan, China.

**Keywords:** congenital heart disease, exercise training, meta-analysis, systematic review, tetralogy of Fallot

## Abstract

**Background:**

The arrival of transcatheter mitral valve therapies has provided feasible and safe alternatives to medical and surgical treatments for mitral regurgitation. The aim of this study is to estimate the relative efficacy and safety of exercise training in patients with corrected tetralogy of Fallot through meta-analysis.

**Methods:**

: A systematic search will be performed using PubMed, EMBASE, the Cochrane Library, Web of Science, CBM, CNKI, WanFang Data, and VIP to include random controlled trials or nonrandom controlled trials comparing the efficacy and safety of exercise training in corrected tetralogy of Fallot patients. The risk of bias for the included nonrandom controlled studies will be evaluated according to Risk of Bias in Nonrandomized Studies of Interventions. We will use the Cochrane Collaboration's tool (version 2 of the Cochrane risk of bias tool for randomized trials) to assess risk of bias of included random controlled trials. Revman 5.4 and STATA 15.0 will be used to complete the meta-analysis and generate forest plots. Grading of recommendations assessment, development, and evaluation will be used to assess the quality of evidence.

**Results:**

: The results of this systematic review and meta-analysis will be submitted to a peer-reviewed journal for publication.

**Conclusion:**

: This study will provide broad evidence of efficacy and safety of exercise training in patients with corrected tetralogy of Fallot and provide suggestions for clinical practice and future research.

**Protocol registration number:**

INPLASY202150006.

## 1 Introduction

The birth prevalence of congenital heart disease (CHD) had gradually risen to the current 9.4 cases per 1000 live births, with an increase of 10% over 10 years ago.^[1,2]^ Improvements in surgical practice and intervention had resulted in a significant reduction in CHD mortality.^[3,4]^ The number of deaths due to CHD in 2017 was estimated with a decrease of 34.5% from the estimated in 1990.^[5]^ The decline in mortality had also led to an estimated 12 million patients with CHD worldwide in 2017, an increase of 18.7% from 1990. In recent years, the long-term prognosis of patients with CHD has been greatly increased. With all improvements in care, it might be predicted that adults with CHD will eventually grow to an estimated 75,000 patients per 10 million residents.^[6]^

Current public health guidelines recommend that children and adults perform moderate to vigorous exercise for ≥60 minutes a day, even in patients with CHD after surgery.^[7–9]^ Exercise-based cardiac rehabilitation is widely used in patients with acquired ischemic heart disease or dilated cardiomyopathy, and has been shown to improve aerobic exercise capacity, relieve symptoms, reduces the long-term risks associated with heart failure, and improve the survival rate of these patients.^[10–12]^ However, many patients with CHD do not exercise. This may be partly due to concerns about the negative effects of exercise on their hearts and the overprotection of these children by their parents. In addition, physicians who care for these patients are reluctant to encourage these patients to exercise, and often ignore the discussion of this issue.^[13–15]^ These patients have limited understanding of adverse cardiac events during exercise and the impact of exercise on cardiac remodeling, which may hinder the implementation of these public health guidelines.

Among all patients with CHD, those with tetralogy of Fallot (ToF) are most likely to develop heart failure, and it can be considered to benefit most from exercise training.^[16,17]^ Patients with ToF can live a relatively normal life and grow to adulthood after surgical repair in childhood.^[18]^ Many studies have reported that in patients with corrected ToF, their exercise capacity, blood vessel, and cardiac autonomic nerve function are still damaged, and their health-related quality of life may be reduced.^[19–21]^ Compared with healthy peers, these patients have lower aerobic exercise ability. Residual cardiopulmonary disease, sedentary, avoidance of physical activity, and a lack of physical activity lifestyle are thought to be responsible for these differences.^[22,23]^ Exercise training can be expected to offset these damages.

Previous studies have compared the effects of exercise on cardiopulmonary function in patients with ToF.^[24–26]^ These original studies show that daily physical exercise in children with ToF has a positive effect, and there is an interaction between daily physical activity and cardiopulmonary adaptation. However, the effects of exercise training on vascular function and cardiac autonomic nervous system are still elusive. It is not clear whether exercise will lead to adverse cardiac remodeling, which will lead to heart enlargement and heart function decline. In addition, the specific effects of different intensities of exercise training are not yet supported by definite synthesis evidence. In this study, we will conduct a systematic review and meta-analysis to evaluate the effectiveness and safety of exercise training on patients with corrected ToF.

## 2 Methods

### 2.1 Protocol and registration

This protocol will be reported according to the Preferred Reporting Items for Systematic Review and Meta-Analysis Protocols (PRISMA-P) 2015 and the checklist is presented in online Supplementary Appendix 1.^[27]^ This protocol was registered with the International Platform of Registered Systematic Review and Meta-Analysis Protocols (INPLASY) on May 1, 2021. The registration number is INPLASY202150006.

### 2.2 Search strategy

We will perform a systematic search via PubMed, EMbase, the Cochrane Library, Web of Science, CBM, CNKI, WanFang Data, and VIP. Besides, the reference lists of included studies and other relevant articles will be retrieved for supplement. Take PubMed which planned to be searched as an example, the presearch strategy is presented as follows:

#1 “Heart Defects, Congenital” [Mesh] OR “Tetralogy of Fallot” [Mesh]
#2 “congenital heart defect” [Title/Abstract] OR “congenital heart disease” [Title/Abstract] OR “heart abnormality” [Title/Abstract] OR “congenital heart defects” [Title/Abstract] OR “congenital heart diseases” [Title/Abstract] OR “heart abnormalities” [Title/Abstract]
#3 (“tetralogy” [Title/Abstract] OR “trilogy” [Title/Abstract] OR “syndrome” [Title/Abstract]) AND “fallot∗” [Title/Abstract]
#4 #1 OR #2 OR #3
#5 “Exercise” [Mesh] OR “Exercise Therapy” [Mesh] OR “Exercise Movement Techniques” [Mesh] OR “Physical Exertion” [Mesh] OR “Physical Fitness” [Mesh] OR “Physical Therapy Modalities” [Mesh] OR “Physical Endurance” [Mesh] OR “Resistance Training” [Mesh] OR “rehabilitation” [Mesh] OR “Walking” [Mesh] OR “Bicycling” [Mesh]
#6 “rehabilitation” [Title/Abstract] OR “exercise∗” [Title/Abstract] OR “physical therap∗” [Title/Abstract] OR “physical training” [Title/Abstract] OR “physiotherap∗” [Title/Abstract] OR “resistance training” [Title/Abstract] OR “fitness” [Title/Abstract] OR “aerobic∗” [Title/Abstract] OR “endurance” [Title/Abstract] OR “treadmill∗” [Title/Abstract] OR “walking” [Title/Abstract] OR “bicycling” [Title/Abstract] OR “quality of life” [Title/Abstract]
#7 #5 OR #6
#8 #4 AND #7

### 2.3 Eligibility criteria

#### 2.3.1 Type of participants

Patients after surgical repair of ToF, regardless of gender and age. Patients with ventricular outflow tract obstruction with a Doppler derived peak > 60 mm Hg were excluded.

#### 2.3.2 Type of intervention

The intervention group was defined as receiving exercise-based cardiac rehabilitation training. According to previous studies, exercise rehabilitation needs to meet 3 times a week, at least 1 hour of planned aerobic exercise each time, and persist for 2 months.^[24,26]^ We do not impose other restrictions on higher-intensity exercise training programs.

#### 2.3.3 Type of comparison

The control group did not receive an exercise-based cardiac rehabilitation training program, and just chose a normal daily lifestyle.

#### 2.3.4 Type of outcomes

The outcomes of our study including cardiopulmonary fitness, disease-specific biomarkers (lipid status, N terminal pro B type natriuretic peptide, and fibrinogen levels), cardiac autonomic function (heart rate variability and post-exercise heart rate recovery), and health-related quality of life.

#### 2.3.5 Type of studies

Randomized and nonrandomized controlled studies will be both included, and related systematic reviews or meta-analysis will be also attended for retrieving their applicable reference.

#### 2.3.6 Other criteria

We will include studies with language of English or Chinese, and there will be no restrictions on the year of publication and publication status.

### 2.4 Study selection

The retrieved articles from the databases will be exported to EndNote X9 Thomson Reuters (Scientific) LLC, Philadelphia, PA) software for duplicate removal and further categorization. Two authors will independently screen the titles and abstracts, than to determine the preliminary inclusion of systematic reviews according to the eligibility criteria. While insufficient data are available or where there is any uncertainty in the abstract, the full-text will be retrieved. Any differences in selection will be resolved through discussion to reach a consensus or by adjudicating with a third reviewer. We will record the excluded articles and the reasons for their exclusion. If necessary, we will get additional information for unclear or doubtful data from the corresponding authors by email.

### 2.5 Data extraction

The reviewers will extract the following data items: Bibliographic details (author, institution, publication year, journal, country, funding). Methodological characteristics (search end date, study design, length of duration of exercise training). Patient characteristics (age, gender, race, time to undergo corrected surgery for ToF). Outcomes (cardiopulmonary fitness, disease-specific biomarkers, cardiac autonomic function, and health-related quality of life). A third author will check the data extracted by the 2 reviewers, and finally reach a consensus on the inconsistent data through discussion.

### 2.6 Risk of bias of individual studies

We assessed risk of bias of included randomized studies according to the Cochrane “Risk of bias” assessment tool version 2, including bias arising from the randomization process, bias due to deviations from intended interventions, bias due to missing outcome data, bias in measurement of the outcome, and bias in selection of the reported result.^[28]^ We will assess risk of bias as low, high, or unclear risk of bias.

The risk of bias of included nonrandomized studies will be assessed according to the tool named Risk Of Bias in Non-randomized Studies-of Interventions, which is divided into 7 domains including bias due to confounding (preintervention), bias in selection of participants into the study (preintervention), bias in classification of interventions (at intervention), bias due to deviations from intended interventions (postintervention), bias due to missing data (postintervention), bias in measurement of outcome (postintervention), and bias in selection of the reported result (postintervention), while finally with an assessment of overall risk of bias.^[29]^ The risk of bias will be evaluated as low, moderate, serious, critical risk of bias, and no information.

The risk of bias assessment will be completed by 2 independent reviewers, and disagreements will be resolved by a third reviewer.

### 2.7 Statistical analysis

#### 2.7.1 Data synthesis

We processed data in accordance with the Cochrane Handbook for Systematic Reviews of Interventions.^[28]^ Revman 5.4 (Nordic Cochrane Centre, Denmark) will be used to complete the meta-analysis and generate forest plots. We expressed dichotomous outcomes as risk ratios, and calculated 95% confidence intervals for each study. For continuous variables, we compared net changes (that is intervention group minus control group differences) and calculated mean difference and 95% confidence intervals for each study. We sought missing data from investigators to obtain key information or missing numerical outcome data where possible. If SDs for outcomes were not reported and were not provided by study authors, then we imputed these values from data within the trial using methods outlined in the Cochrane Handbook for Systematic Reviews of Interventions.

#### 2.7.2 Assessment of heterogeneity

For each outcome, we carried out tests of heterogeneity using the *χ*^2^ test of heterogeneity and the I^2^ statistic. Where no or minimal heterogeneity was present, we performed fixed-effect model meta-analyses. Where substantial heterogeneity was detected (I^2^≥ 50%), we evaluated the results for possible explanations (for example participants and interventions) and performed random-effects model meta-analysis with cautious interpretation.

#### 2.7.3 Subgroup analysis and sensitivity analysis

We need to solve heterogeneity because it is only when the included studies have the least heterogeneity, the credibility of the synthesized effect size is high, and sensitivity and subgroup analyses are the most common approaches used to solve heterogeneity. If the results of meta-analysis are positive and the number of included studies is over 3, we will analyze the sensitivity using STATA 15.0 software. The sensitivity analysis is performed by excluding study one by one. The sensitivity is low and the results are of stability and reliability if there are no significant changes that appear in the results before and after the exclusion; if not, it indicates a high sensitivity and unstable result. And in this study, year of publication, country of corresponding author, type of study design, mean age, training intensity, and length of exercise-training time will be considered and designed for subgroup analysis to find the possible significant heterogeneity.

### 2.8 Quality of evidence

We will use the grading of recommendations assessment, development, and evaluation (GRADE) approach to evaluate the quality of the evidence.^[30]^ The GRADE approach has considerations of 5 aspects, including study limitations, consistency of effect, imprecision, indirectness, and publication bias, for the evaluation of the quality of the body of evidence about each outcome. It is categorized as 4 levels: high level, moderate level, low level, and very low level.

### 2.9 Patient and public involvement

Patients or the public will not be involved in the design, conduct, reporting, or dissemination plans of our research.

## 3 Discussion

Our study will compare the safety and effectiveness of exercise training for patients with repaired ToF, evaluate the impact of exercise on cardiac remodeling, and provide high-level evidence support for current clinical guidelines. For patients with corrected ToF, moderate exercise training can improve aerobic capacity. We should encourage patients to receive regular exercise. Whether it is high-intensity interval training or medium-intensity continuous training, it is safe for patients with repaired ToF. Most importantly, exercise training seems to improve at least certain parameters of cardiovascular health.^[31–33]^ These conclusions require rigorous evidence evaluation to verify internal authenticity and external promotion. For all we have known, this study will first make a systematic review and meta-analysis to verify the above conclusions, so as to better promote the dissemination of current guidelines.

This systematic review and meta-analysis will summarize the direct and indirect evidence to evaluate and compare the safety and effectiveness of exercise training for patients with corrected ToF. Furthermore, we will evaluate the risk of bias of each included study via Cochrane Risk of bias assessment tool version 2 or risk of bias in nonrandomized studies of interventions, and assess the quality of evidence using the GRADE framework. We hope that our study will provide suggestions for clinical practice and future research.

## Acknowledgments

The authors gratefully acknowledge the financial supports by the Natural Science Foundation of Gansu Province (project number: 21JR1RA027) and Health industry scientific research project of Gansu Province (project number GSWSKY2016-04).

## Author contributions

**Conceptualization:** Ya-Qin Gong, Xiao-Yan Liu.

**Data curation:** Ya-Qin Gong, Xiao-Yan Liu, Ping Zhi, Fang-Fei Guo, Yun-Xia Wang.

**Formal analysis:** Ya-Qin Gong, Xiao-Yan Liu, Li-Na Wei, Jin-Zhi Qian, He-Li Dong.

**Funding acquisition:** Ya-Qin Gong.

**Investigation:** Ya-Qin Gong, Xiao-Yan Liu, Ping Zhi, Fang-Fei Guo, Yun-Xia Wang, He-Li Dong.

**Methodology:** Ya-Qin Gong, Xiao-Yan Liu, Li-Na Wei, Jin-Zhi Qian.

**Supervision:** Ya-Qin Gong, Xiao-Yan Liu.

**Writing – original draft:** Ya-Qin Gong, Xiao-Yan Liu.

**Writing – review & editing:** Ya-Qin Gong, Xiao-Yan Liu, Ping Zhi, Li-Na Wei, Fang-Fei Guo, Jin-Zhi Qian, Yun-Xia Wang, He-Li Dong.

## Supplementary Material

Supplemental Digital Content
